# Soluble Fraction from Lysate of a High Concentration Multi-Strain Probiotic Formulation Inhibits TGF-β1-Induced Intestinal Fibrosis on CCD-18Co Cells

**DOI:** 10.3390/nu13030882

**Published:** 2021-03-09

**Authors:** Francesca Lombardi, Francesca Rosaria Augello, Paola Palumbo, Elona Mollsi, Maurizio Giuliani, Anna Maria Cimini, Maria Grazia Cifone, Benedetta Cinque

**Affiliations:** 1 Department of Life, Health & Environmental Sciences, University of L’Aquila, Via Pompeo Spennati, Building Rita Levi Montalcini, Coppito, 67100 L’Aquila, Italy; francesca.lombardi@univaq.it (F.L.); francescarosaria.augello@graduate.univaq.it (F.R.A.); paola.palumbo@univaq.it (P.P.); elona.mollsi@graduate.univaq.it (E.M.); maurizio.giuliani@univaq.it (M.G.); annamaria.cimini@univaq.it (A.M.C.); mariagrazia.cifone@univaq.it (M.G.C.); 2 Unit of Plastic and Reconstructive Surgery, Casa di Cura Di Lorenzo SrL, Via Vittorio Veneto 37, Avezzano, 67051 L’Aquila, Italy; 3 Department of Biology, Sbarro Institute for Cancer Research and Molecular Medicine, Temple University, Philadelphia, PA 19019, USA

**Keywords:** intestinal fibrosis, CCD-18Co cells, TGF-β1, VSL#3^®^, Vivomixx^®^, collagen-I, α-SMA, Smad 2/3

## Abstract

Fibrosis is a severe complication of chronic inflammatory disorders, such as inflammatory bowel disease (IBD). Current strategies are not fully effective in treating fibrosis; therefore, innovative anti-fibrotic approaches are urgently needed. TGF-β1 plays a central role in the fibrotic process by inducing myofibroblast differentiation and excessive extracellular matrix (ECM) protein deposition. Here, we explored the potential anti-fibrotic impact of two high concentration multi-strain probiotic formulations on TGF-β1-activated human intestinal colonic myofibroblast CCD-18Co. Human colonic fibroblast CCD-18Co cells were cultured in the presence of TGF-β1 to develop a fibrotic phenotype. Cell viability and growth were measured using the Trypan Blue dye exclusion test. The collagen-I, α-SMA, and pSmad2/3 expression levels were evaluated by Western blot analysis. Fibrosis markers were also analyzed by immunofluorescence and microscopy. The levels of TGF-β1 in the culture medium were assessed by ELISA. The effects of commercially available probiotic products VSL#3^®^ and Vivomixx^®^ were evaluated as the soluble fraction of bacterial lysates. The results suggested that the soluble fraction of Vivomixx^®^ formulation, but not VSL#3^®^, was able to antagonize the pro-fibrotic effects of TGF-β1 on CCD-18Co cells, being able to prevent all of the cellular and molecular parameters that are related to the fibrotic phenotype. The mechanism underlying the observed effect appeared to be associated with inhibition of the TGF-β1/Smad signaling pathway. To our knowledge, this study provides the first experimental evidence that Vivomixx^®^ could be considered to be a promising candidate against intestinal fibrosis, being able to antagonize TGF-β1 pro-fibrotic effects. The differences that were observed in our fibrosis model between the two probiotics used could be attributable to the different number of strains in different proportions.

## 1. Introduction

Intestinal fibrosis is the more detrimental feature of inflammatory bowel disease (IBD). It is mainly characterized by excessive extracellular matrix (ECM) protein deposition that is caused by abnormal activation of myofibroblasts, which are considered the crucial effector cells of the fibrotic process [1,2,3]. The overactivated myofibroblasts derive from multiple cell types, including subepithelial fibroblasts, smooth muscle cells, and via epithelial-mesenchymal or endothelial-mesenchymal transition. A central signature of myofibroblast differentiation is the change in shape, which is characterized by the highly structured scaffold of actin stress fibers and intermediate filaments able to increase cell mobility and facilitate cell activation. Although the pathogenesis of IBD-associated fibrostrictures is not fully understood, accumulating evidence suggests that inflammation may no longer be required to sustain fibrogenesis in the late stages of the disease. Fibroblasts can secrete various pro-inflammatory cytokines, which target adjacent stromal cells and activate multiple signaling pathways, thus increasing further ECM deposition and perpetuating the fibrotic process [3,4,5].

TGF-β1 represents an essential mediator in the pro-fibrotic signaling, playing a central role in the myofibroblast activation, thus promoting ECM synthesis and inhibiting its degradation [6,7]. The canonical TGF-β1 pathway includes the Smad2/3 phosphorylation, which promotes the expression of specific genes for collagens, fibronectin, and plasminogen activator inhibitor-1 [8].

To date, no specific intestinal anti-fibrotic therapy exists, nor has any immunosuppressive or biologic drug been shown to prevent stricture formation. Indeed, the standard anti-fibrotic approach mainly focuses on repressing inflammation; however, a lot of evidence suggests that it cannot slow down disease progression or reverse fibrosis established by now [9]. The surgery for bowel resection is the therapeutic option in more than two-thirds of Crohn’s disease (CD) patients for symptomatic fibrostrictures, with high recurrence along with medically refractory disease [10]. A surgical procedure is currently the only choice to relieve intestinal fibrotic complications, but it often provides short-term benefits. Therefore, it is crucial to effectively develop new alternative treatments to target the initiation and progression of intestinal fibrosis.

The effects of probiotics and/or probiotic lysates in controlling intestinal fibrosis are still mostly unknown. The data available are still preliminary and they need to be confirmed and expanded. A study reported that polyphosphate, a bioactive molecule that is produced by *Lactobacillus brevis,* improved the intestinal inflammation and fibrosis in two murine colitis models and reduced inflammatory cytokine expression in colonic epithelial cells, but it did not influence collagen expression in the CCD-18Co cells [11]. Park et al. (2018) demonstrated that administering a complex of 12 probiotics or their combination with prebiotics, rosavin, and zinc in a colitis mouse model dampened colitis and improved colitis-associated fibrosis. A significant reduction of α-SMA and collagen-I positive cells was detectable in colonic sections [12]. Recently, a new probiotic strain, *L. lactis* ML2018, identified for anti-inflammatory properties, has been evaluated in the dextran sulfate sodium (DSS)-induced murine model of colitis, leading to colon fibrosis [13]. The obtained data demonstrated that the oral administration of *L. lactis* ML2018 reduced the levels of several colon fibrosis markers, showing a significant protective effect in the colon tissue of mice that were exposed to DSS. Deng et al. (2020) studied the impact of a combination of four different probiotics on abdominal adhesions that are caused by surgery in a rat model [14]. The probiotic treatment downregulated the TGF-β1/Smad signaling pathway. The consequence was a significant reduction of pro-inflammatory cytokine levels, inflammatory cellular infiltration, and tissue fibrosis.

Colonic intestinal fibroblast cells (CCD-18Co) that are activated in vitro by TGF-β1 represent a widely intestinal fibrosis model used in experimental studies [15,16]. In the present work, we aimed to investigate the effects of the soluble fraction of lysates from two different high concentration multi-strain probiotic formulations (VSL#3^®^ and Vivomixx^®^) on the TGF-β1-induced fibrotic process in CCD-18Co fibroblasts. The results show that Vivomixx^®^-derived lysate fraction inhibited collagen-I and α-SMA expression in human colonic fibroblast cell line CCD-18Co by interfering with TGF-β1/Smad2/3 signaling while VSL#3 was not active.

## 2. Materials and Methods

### 2.1. Soluble Fractions from Bacterial Lysate Preparation for Cell Treatments

VSL#3^®^ Capsules, which were manufactured in Italy (distributed by Ferring Pharmaceuticals S.p.A., Milan, Italy) 0.658 g, according to the information on the boxes, containing 112 billion Colony Forming Units (CFU): *Streptococcus thermophilus* BT01, *Bifidobacterium breve* BB02, *Bifidobacterium longum* BL03^§^, *Bifidobacterium infantis* BI04^§^, *Lactobacillus acidophilus* BA05, *Lactobacillus plantarum* BP06, *Lactobacillus paracasei* BP07, *Lactobacillus debrueckii* subsp. *bulgaricus* BD08^§§^ (^§^Reclassified as *Bifidobacterium lactis*; ^§§^reclassified as *Lactobacillus helveticus).* The VSL#3^®^ product that was used in this study is different from the De Simone Formulation that was available under the same VSL#3^®^ trademark until early 2016. Accordingly, all the studies performed before 2016 cannot be referent to the present VSL#3^®^.

Vivomixx^®^ Capsules manufactured in the USA (trade name Vivomixx^®^ in EU, Visbiome^®^ in USA, De Simone Formulation^®^ in Korea) (distributed by Biospaera Pharma S.r.L.) 0.676 g, according to the information on the boxes containing 112 billion CFU: *Streptococcus thermophilus* DSM 24731, *Bifidobacterium breve* DSM 24732, *Bifidobacterium longum* DSM 24736*, *Bifidobacterium infantis* DSM 24737*, *Lactobacillus acidophilus* DSM 24735, *Lactobacillus plantarum* DSM 24730, *Lactobacillus paracasei* DSM 24733, and *Lactobacillus delbrueckii* subsp. *bulgaricus* DSM 24734** (*reclassified as *Bifidobacterium lactis*; **reclassified as *Lactobacillus helveticus*).

For bacterial fraction preparations, the content of each capsule was suspended at the concentration of 102 × 10^9^ CFU in 10 mL of Phosphate Buffered Saline (PBS, Euro Clone, West York, UK), centrifuged at 8600× g, washed twice, and then sonicated (30 min, alternating 10 s of sonication and 10 s of pause) using a Vibracell sonicator (Sonic and Materials, Danbury, CT, USA). Bacterial cell disruption was verified by measuring the sample’s absorbance at 590 nm (Eppendorf Hamburg, Germany) before and after every sonication step. The samples were then centrifuged at 17,949× *g*. The supernatants were filtered using a 0.22-µm-pore filter (Corning Incorporated, Corning, NY, USA) to remove any whole bacteria remaining and obtaining the soluble fraction of bacterial lysates from both probiotic formulations. The total protein content was determined by DC Protein Assay (BioRad, Hercules, CA, USA) while using bovine serum albumin (BSA, Sigma Aldrich, Saint Louis, MO, USA) as standard. According to our previous experience [17,18], for all of the below described in vitro cell treatments, bacterial fractions were prepared to obtain a final concentration of 50 µg protein/mL, equivalent to 10^8^ CFU/mL.

### 2.2. Cell Line and Culture Conditions

Human intestinal CCD-18Co fibroblast cell line (ATCC CRL-1459) was obtained from American Type Culture Collection (ATCC, Georgetown, DC, USA) and then cultured in DMEM that was supplemented with 10% of Fetal Bovine Serum (FBS), 100 U/ml penicillin, 100 mg/ml streptomycin, and 2 mM glutamine. The cell cultures were maintained in a humidified atmosphere of 95% air and 5% CO_2_ at 37 °C. Where not otherwise specified, the reagents for cell biology and consumables were purchased from EuroClone (EuroClone, West York, UK). After reaching 80% confluence, the cells were sub-cultured; the experiments were carried out at the 15th passage.

### 2.3. Cell Viability and Growth

After treatments, the cells were harvested and centrifuged for 10 min at 400× *g*. The pellets were incubated with a 0.04% Trypan blue (Euro Clone, West York, UK) solution for 5 min. to analyze cell number and viability. Not-treated cells were also analyzed and they served as controls. The cells were transferred to a Bürker counting chamber and then counted by microscopy (Eclipse 50i, Nikon Corporation, Tokyo, Japan).

For all of the experiments, the CCD-18Co cells were plated at 5000 cells/cm^2^, grown overnight, and then serum-deprived for 24 h before the addition of indicated stimuli in the serum-free medium. The CCD-18Co cells were stimulated for 48 h with 10 ng/mL human transforming growth factor (hTGF-β1; Cell signaling Technology, MA, USA) to induce the fibrotic phenotype. The cells were stimulated with TGF-β1 (10 ng/mL) for 48 h in the presence or absence of bacterial fractions from VSL#3^®^ or Vivomixx^®^ lysates (50 µg protein/mL corresponding to 10^8^ CFU/mL) to investigate the effects of probiotic lysate-derived fractions. After treatments, the cells were harvested and then centrifuged for 10 min. at 400× *g.* The pellets were incubated with a 0.04% Trypan blue (Euro Clone, West York, UK) solution for 5 min. to analyze cell number and viability. Not treated cells served as controls. The cells were transferred to a Bürker counting chamber and then counted by microscopy (Eclipse 50i, Nikon Corporation, Tokyo, Japan).

### 2.4. Western Blot Analysis

For Western blot analyses, cell pellets were collected, washed in PBS, and then lysed in RIPA buffer (Merck KGaA, Darmstadt, Germany) containing a protease inhibitor mixture (carboxypeptidase inhibitor, 5 μg/mL trypsin inhibitor, 1 mM PMSF, 10 μg/mL leupeptin, 10 μg/mL aprotinin, and 10 μg/mL pepstatin) (Sigma Aldrich, St. Louis, MO, USA). The samples were assayed for protein content with DC Protein Assay (BioRad, Hercules, CA, USA) using BSA as standard. 25 μg of proteins were mixed with sample buffer, boiled for 5 min. at 100 °C, and then separated by 10% SDS-polyacrylamide gel electrophoresis. The proteins were transferred onto 0.45 µm nitrocellulose membrane sheets (BioRad) for 1 h at 4 °C at 70 V using a Mini Trans-Blot Cell apparatus (BioRad). Membranes were blocked with 5% non-fat dry milk for one hour at room temperature and then incubated overnight at 4 °C with mouse monoclonal antibody anti-α-actin smooth muscle (ACTA2, α-SMA) (OriGene, Rockville, MA, USA) 1:1000, or with rabbit polyclonal antibody anti-COL1A1, (Boster Biological Technology, Pleasanton, CA, USA) 1:1000, or with rabbit monoclonal antibody anti-human phospho-Smad2/3 (phospho-S465/S467), (R&D Systems Inc., Minneapolis, MN, USA) 1:100, or with rabbit monoclonal phospho-Nrf2 (phospho-S40), (Abcam, Cambridge, UK) 1:2000, and mouse monoclonal antibody anti-GAPDH (Origene, 9620 Medical Center Drive Suite 200 Rockville, MD, USA) 1:1000. Horseradish peroxidase (HRP)-conjugated goat anti-rabbit IgG secondary antibody at 1:2000 was used for anti-COL1A1, anti-human phospho-Smad2/3, and anti-phospho-Nrf2 antibodies, and horseradish peroxidase (HRP)-conjugated rabbit anti-mouse IgG secondary antibody at 1:2000 for anti-α-SMA and anti-GAPDH antibodies (Millipore EMD, Darmstadt, Germany). The immuno-reactive bands were visualized by enhanced chemiluminescence (ECL, Amersham Pharmacia Biotech), according to the manufacturer’s instructions. Band relative densities were determined using a chemiluminescence documentation system ALLIANCE (UVITEC, Cambridge UK), and the values were given as relative units. Immunoblot data were normalized to the relative GAPDH bands.

### 2.5. Immunofluorescence Staining for Fibrotic Markers

CCD-18Co cells that were grown on coverslips in a 12-well plate (seeded at 5000 cells/cm^2^) were treated as reported above for the indicated time points. The coverslips were then washed with PBS, fixed with 4% formaldehyde for 20 min., permeabilized with 0.1% Triton X-100 (Sigma-Aldrich, St. Louis, MO, USA) for 5 min., and blocked with 3% BSA (Sigma–Aldrich) for 20 min. at room temperature. Subsequently, cells were incubated overnight at 4 °C with rabbit polyclonal antibody anti-COL1A1 (Boster Biological Technology, Pleasanton, CA, USA) 1:250, or mouse monoclonal antibody anti-α-actin smooth muscle (ACTA2, α-SMA) (OriGene, Rockville, MA, USA) 1:250. Subsequently, the coverslips were stained using a FITC conjugated goat anti-rabbit polyclonal IgG secondary antibody (Millipore EMD, Darmstadt, Germany) 1:1000 or FITC conjugated goat anti-mouse polyclonal IgG secondary antibody (Bethyl Laboratories, Inc, Montgomery, TX, USA) for 1 h at room temperature, washed, and then incubated with TRITC labeled phalloidin (Sigma-Aldrich) for 45 min. at room temperature. The coverslips were mounted with VECTASHIELD^®^ Antifade Mounting Medium with DAPI (Vector Laboratories, Inc., Burlingame, CA, USA) and then examined at 10× and 40× magnifications with a fluorescent microscope (Eclipse 50i, Nikon, Tokyo, Japan).

### 2.6. TGF-β1 ELISA

The levels of released TGF-β1 were quantified in the cell supernatants using a human TGF-β1 enzyme-linked immunosorbent assay (ELISA) kit (Sigma Aldrich, Saint Louis, MO, USA), as described in the manufacturer’s instructions and expressed as pg/mL. Briefly, the CCD-18Co cells were plated at 5000 cells/cm^2^, grown overnight, and then serum-deprived for 24 h. After the cells were incubated with or not TGF-β1 (10 ng/mL) for 48 h, in the presence or absence of bacterial fraction from VSL#3^®^ or Vivomixx^®^ (50 µg protein/mL) in a serum-free medium. After treating the cells, the media were collected, cleared of cellular debris/dead cells by centrifugation at 600× *g* for 10 min., and the TGF-β1 concentration was then determined in the medium using an ELISA kit.

### 2.7. Statistical Analysis

The data were analyzed using Prism 6.0 (GraphPad Software, San Diego, CA, USA). A one-way ANOVA or a two-way ANOVA, followed by Bonferroni post hoc test, was used to compare the mean values among groups. We accepted the values of *p* < 0.05 as significant. 

## 3. Results

### 3.1. Effects of the Bacterial Fractions from VSL#3^®^ and Vivomixx^®^ on TGF-β1-Induced CCD-18Co Cell Proliferation

**Figure 1:**
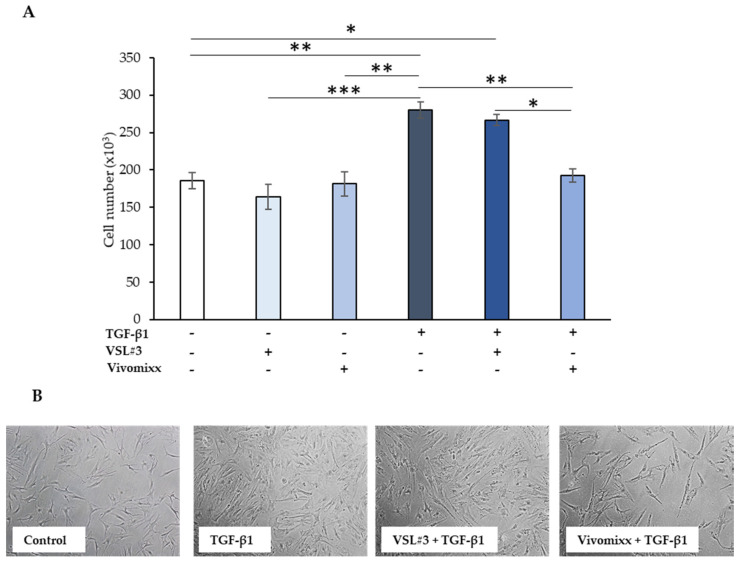
Effect of the soluble fraction of bacterial lysates from VSL#3 and Vivomixx formulations on colonic intestinal fibroblast cells (CCD-18Co) cell number. (**A**) CCD-18Co cells were starved for 24 h and then incubated with TGF-β1 (10 ng/mL) for 48 h in the presence or absence of VSL#3- or Vivomixx-derived fractions (50 µg/mL), in serum-free medium. The values represent the cell number expressed as mean ± SEM of three independent experiments in duplicate. For comparative analysis of data groups, a one-way analysis of variance (ANOVA) with post hoc Bonferroni test was used. * p < 0.05, ** *p <* 0.01, *** *p* < 0.001. (**B**) Representative phase-contrast microscopic images showing CCD-18Co fibroblasts treated as described in (**A**).

As expected, and confirming previous reports [19], treatment with TGF-β1, at 10 ng/mL for 48 h, enhanced CCD-18Co cell proliferation (~50% than the untreated control cells, *p* < 0.01), as shown in Figure 1A. Both bacterial fractions alone in the culture medium did not significantly affect either vitality or cell proliferation rate, which were kept similar to the basal level of control cells. Of note, while the soluble fraction of lysates from VSL#3^®^ formulation did not influence the TGF-β1-induced cell growth rate, the mean number of cells that were treated with Vivomixx^®^-derived fraction plus TGF-β1 at 48 h was significantly lower either with respect to TGF-β1 alone (*p* < 0.01) or TGF-β1 plus VSL#3^®^-derived fraction (*p <* 0.05) and it was comparable to basal levels (Figure 1A). The cell number counting results were consistent with those that were obtained by phase-contrast microscopic images (Figure 1B). The lower cell density in the Vivomixx^®^-fraction treated sample was evident as compared to both of those stimulated for 48 h with TGF-β1 and the one treated for the same time with the combination TGF-β1 plus VSL#3^®^-derived fraction.

### 3.2. VSL#3^®^- and Vivomixx^®^-Derived Fractions Differently Affect Fibrotic Markers in TGF-β1-Activated CCD-18Co Cells

**Figure 2:**
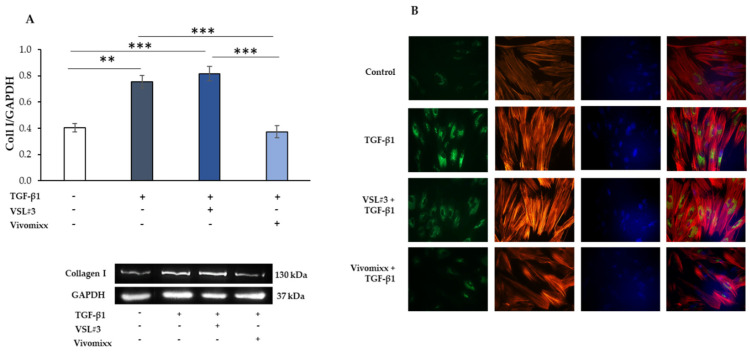
Influence of the soluble fraction from VSL#3 and Vivomixx lysates on TGB-β1-induced collagen I expression in CCD-18Co cells. (**A**) Immunoblotting assay for collagen I was performed on CCD-18Co cells incubated for 48 h with TGF-β1 (10 ng/mL) in the presence or absence of VSL#3- or Vivomixx-derived fraction (50 µg/mL). Following densitometric analysis, obtained values were normalized vs. GAPDH. Data are from three independent experiments in duplicate, and values are expressed as mean ± SEM. For comparative analysis of data, a one-way analysis of variance (ANOVA) with post hoc Bonferroni test was used. ** *p <* 0.01, *** *p <* 0.001. A representative immunoblot of collagen I and GAPDH is also shown. (**B**) Representative immunofluorescence images of CCD-18Co cells treated as described in (**A**) and stained with anti-collagen I antibody (green) and with TRITC-phalloidin (red) to reveal F-actin. Nuclei were counterstained with DAPI (blue) (magnification 40×). The images are representative of three independent experiments in duplicate.

The myofibroblast differentiation represents a key event in intestinal fibrosis’s pathogenesis. It is mainly characterized by a relevant cytoskeleton organization and it increased the expression of significant fibrotic markers, such as collagen-I (Coll I) and the α-SMA protein. Figure 2 shows the results of representative western blot experiments to evaluate the influence of the soluble fraction from lysates from VSL#3^®^ or Vivomixx^®^ formulations of Coll I expression levels. As expected, CCD-18Co cells, serum-deprived for 24 h and then stimulated with TGF-β1 (10 ng/mL) for 48 h, underwent a phenotypic transformation into activated myofibroblast, which was characterized by a significant increase of Coll I (** *p <* 0.01) expression when compared to the control. Of note, the treatment with Vivomixx^®^-derived fraction markedly reduced TGF-β1-induced Coll I expression (*** *p <* 0.001). In contrast, the VSL#3^®^-derived fraction was unable to affect the expression levels of Coll I. These results were confirmed by immunofluorescence analysis. The cells that were treated with TGF-β1 plus VSL#3^®^-derived fraction showed more intense and widespread Coll I stain, similar to those observed with the cells treated with TGF-β1 alone, as observed in the representative images reported in Figure 2B. On the other hand, the Vivomixx^®^ bacterial fraction induced a substantial reduction of staining for Coll I, so much so that the relative fluorescent signals appeared to be weak and diffuse similarly to the control untreated cells.

**Figure 3:**
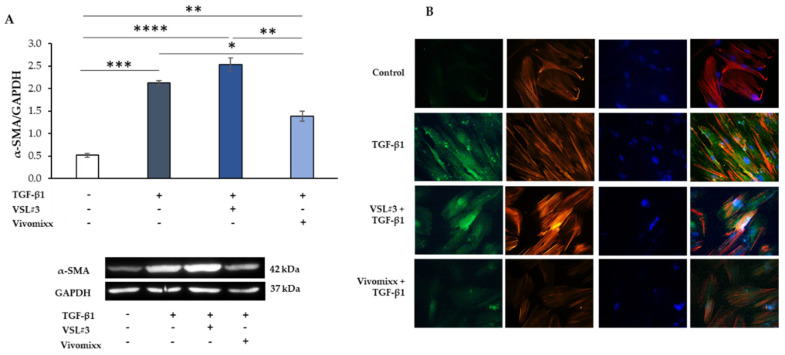
Influence of the soluble fraction from VSL#3 and Vivomixx lysates on TGB-β1-induced α-SMA expression in CCD-18Co cells. (**A**) Immunoblotting assay for α-SMA was performed on CCD-18Co cells incubated for 48 h with TGF-β1 (10 ng/mL) in the presence or absence of VSL#3- or Vivomixx-derived fraction (50 µg/mL). Following densitometric analysis, obtained values were normalized vs. GAPDH. Data are from three independent experiments in duplicate, and values are expressed as mean ± SEM. For comparative analysis of data, a one-way analysis of variance (ANOVA) with post hoc Bonferroni test was used. * *p* < 0.05, ** *p* < 0.01, *** *p* < 0.001, **** *p* < 0.0001. A representative immunoblot of α-SMA and GAPDH is also shown. (**B**) Representative immunofluorescence images of CCD-18Co cells treated, as described in A and stained with anti-α-SMA antibody (green) and with TRITC-phalloidin (red) to reveal F-actin. Nuclei were counterstained with DAPI (blue) (magnification 40×). The images are representative of three independent experiments in duplicate.

Moreover, the fibrotic activation that was induced by TGF-β1 was also characterized by the development of actin stress fibers with remarkable cytoskeletal rearrangement, as shown by phalloidin staining. The presence of Vivomixx^®^ derived fraction dampened the TGF-β1-activated morphology in the cells that indeed appeared with a poorly organized cytoskeleton that was comparable to control cells. On the contrary, when the VSL#3^®^ bacterial sample was added in the presence of TGF-β1, the CCD-18Co cells exhibited a more differentiated morphology with the formation of pronounced actin stress fibers. Similar results were obtained from the experiments analyzing the effects of bacterial fractions of α-SMA expression that was induced in TGF-β1-activated CCD-18Co cells (Figure 3). Once more, the Vivomixx^®^-derived fraction, but not the VSL#3^®^ one, significantly inhibited (* p < 0.05) the expression of the fibrotic marker α-SMA that was induced by TGF-β1 in myofibroblasts, as assessed either by western blot and immunofluorescence analysis (Figure 3A,B). Of note, the VSL#3^®^- and Vivomixx^®^-derived fractions were not able to induce any effects on Coll I and α-SMA expression and cytoskeletal rearrangement, when added in CCD-18Co cells without TGF-β1.

### 3.3. Effects of Probiotic-Derived Fractions on TGF-β1-Related Signaling

**Figure 4:**
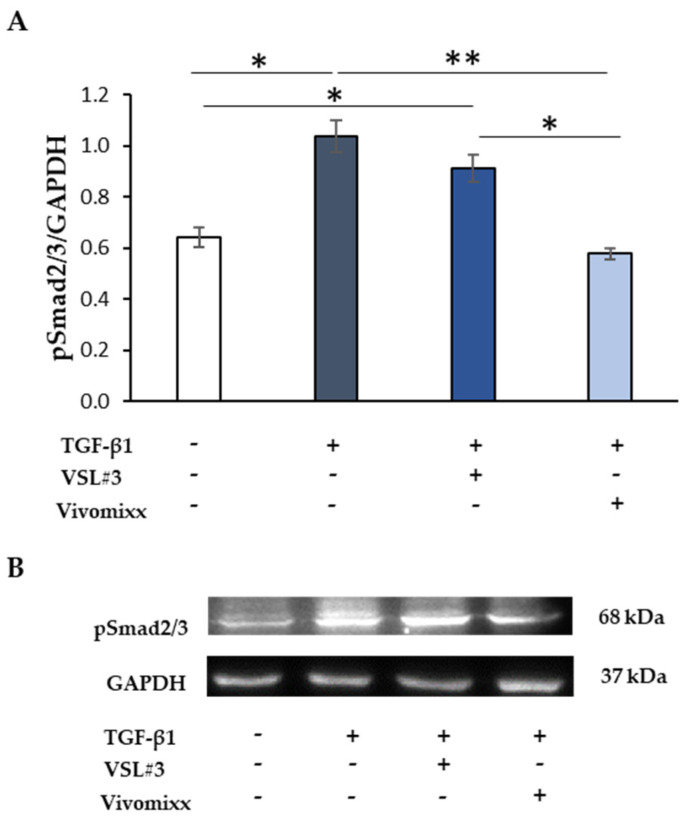
The effect of the soluble fraction from VSL#3 and Vivomixx lysates on TGF-β1-induced Smad activation. (**A**) Immunoblotting assay for pSmad2/3 protein expression was performed on CCD-18Co cells incubated for 48 h with TGF-β1 (10 ng/mL) in the presence or absence of VSL#3- or Vivomixx-derived fractions (50 µg/mL). Following densitometric analysis, the obtained values were normalized vs. GAPDH. The data are from two independent experiments in duplicate, and values are expressed as mean ± SEM. For comparative analysis of data, a one-way analysis of variance (ANOVA) with post hoc Bonferroni test was used. * *p <* 0.05, ** *p* < 0.01. (**B**) A Representative image of immunoblotting for pSmad2/3 and GAPDH is shown.

In the canonical pathway, TGF-β1 is known to induce the Smad signaling for controlling collagen deposition and fibrogenesis [6,20]. Thus, in our experimental conditions, the treatment with TGF-β1 of CCD-18Co cells induced high phosphorylated form levels of Smad2/3 at 48 h (Figure 4). The simultaneous addition of Vivomixx^®^-derived fraction did prevent the effect that was caused by TGF-β1, thus keeping Smad2/3 phosphorylation levels like those of the untreated control. On the contrary, the treatment with VSL#3^®^-derived fraction failed to affect the Smad2/3 phosphorylation, confirming its lack of efficacy as an anti-fibrotic agent.

**Figure 5:**
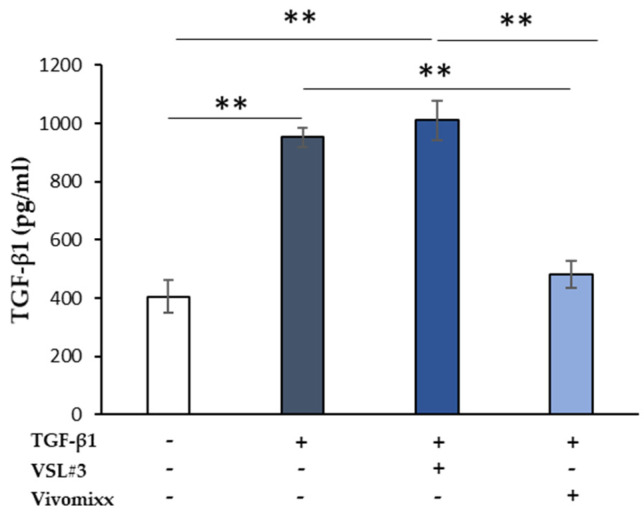
The effect of soluble fraction from VSL#3 or Vivomixx lysates on TGF-β1 secretion in activated CCD-18Co cells. TGF-β1 levels were detected by ELISA in supernatants from CCD-18Co cells that were treated for 48 h with TGF-β1 (10 ng/mL) in the presence or absence of VSL#3- or Vivomixx-derived fractions (50 µg/mL). The results, relative to one representative out of two experiments performed in triplicate, are expressed as mean ± SD. For comparative analysis of data groups, one-way ANOVA followed by post hoc test Bonferroni was used (** *p <* 0.01).

Besides, the levels of TGF-β1 released in the extracellular medium by CCD-18Co cells increased significantly by the addition of TGF-β1 (** *p <* 0.01), likely through an autocrine mechanism (Figure 5). Notably, the addition of Vivomixx^®^-derived fraction, together with TGF-β1, led to a significantly lower TGF-β1 release (** *p <* 0.01) when compared with those observed when the cells were treated with TGF-β1 alone or VSL#3^®^-derived fraction plus TGF-β1. No effect on Smad2/3 phosphorylation and TGF-β1 neosynthesis was observed when VSL#3^®^- and Vivomixx^®^-derived fractions were added alone.

Globally, these results correlated with the ability of Vivomixx^®^-derived fraction well, but not that derived from VSL#3^®^, to significantly inhibit expression of fibrotic markers collagen I and α-SMA, being able to inhibit TGF-β1-triggered signaling in CCD-18Co cells and, consequently, the appearance of the fibrotic phenotype.

## 4. Discussion

In the present work, we evaluated the ability of soluble fraction from bacterial lysates from VSL#3^®^ and Vivomixx^®^ formulations to modulate the TGF-β1 induced-myofibroblast activation in an intestinal fibrosis model in vitro. TGF-β1, a pluripotent cytokine implicated in many cellular and immunological processes, represents the critical activator that is central to the fibrosis process. It regulates numerous genes that are involved in the increased deposition of collagen and other extracellular matrix proteins with progressive tissue distortion, dysfunctional wound healing, and luminal narrowing [6].

The more detrimental feature in IBD is represented by the chronic activity of mucosal inflammatory cells, being able to induce the fibroblast differentiation into myofibroblasts. These myofibroblasts have a contractile shape and the ability to produce great quantities of ECM components. They express specific cellular markers with an intermediate phenotype between fibroblasts and smooth muscle cells. In our experimental conditions, TGF-β1 induced the differentiation of CCD-18Co in myofibroblasts, as evidenced by immunofluorescence and western blot experiments showing a significant overexpression of collagen I and α-SMA. The actin cytoskeleton’s reorganization resulted in thick actin bundles and increased fluorescent staining with TGF-β1 treatment. The untreated cells exhibited a small network of parallel stress fibers confirming the absence of myofibroblast activation. The upregulation of fibrotic markers, i.e., collagen-I and α-SMA, and the cytoskeleton’s remodeling was reverted by the Vivomixx^®^ lysates, which prevented the TGF-β1 induced differentiation of CCD-18Co toward activated myofibroblast. On the contrary, the soluble fraction derived from VSL#3^®^ kept the TGF-β1-induced fibrotic phenotype stable, either in terms of expression levels of collagen-I and α-SMA or the cytoskeletal rearrangement. Consistent with the previous results, the activated form of Smad2/3 significantly increased in TGF-β1-treated CCD-18Co cells. Another critical feature of TGF-β1 is the ability to sustain itself through an autocrine loop inducing its release from fibroblast [21]. TGF-β1-neosynthesis in activated-CCD-18Co cells was significantly stimulated by the addition of TGF-β1; however, the Vivomixx^®^ lysates significantly suppressed the stimulation. Notably, the anti-fibrotic effects only showed by the Vivomixx^®^-derived fraction could be associated with its ability to antagonize either the Smad2/3 phosphorylation or TGF-β1-neosynthesis by activated-CCD-18Co cells.

The ability of probiotics and probiotic-derived functional factors in promoting and maintaining intestinal homeostasis and the potential therapeutic of probiotics in IBD patients by modulating inflammatory processes is well known. On the other hand, few studies are available regarding their effects in IBD-associated intestinal fibrosis. According to the current state of knowledge, probiotic bacteria administration has shown efficacy as an adjuvant therapy in attenuating liver fibrosis [22] as well as the respiratory and gastrointestinal outcomes in a stable cystic fibrosis clinic population [23]. In the same populations, significant alterations in the gut microbiome structure have also been observed [22,23]. These data suggest that probiotics might also play a beneficial role directly in fibrotic tissue that is associated with intestinal inflammatory disease, such as CD. Despite the evidence in favor of the use of probiotics in ulcerative colitis and pouchitis [24,25,26], the data in CD are scant and controversial [27]. 

The present study’s findings suggest that the soluble fraction of lysate from Vivomixx^®^ formulation suppressed the activation and prevented intestinal fibrosis on CCD-18Co cells, which may be associated with an inhibition of the TGF-β1/Smad signaling pathway. It will be important to identify and purify the Vivomixx^®^-derived factor(s) able to reverse the intestinal fibrosis process. It will also be interesting to record the differences between Vivomixx^®^ lysates and VSL#3^®^ lysates concerning active components.

Regarding the differences that were observed between the two probiotic mixtures used, we refer to what was published previously [17,18,28,29,30,31]. The two products are different for composition and manufacturing processes. Variables in the manufacturing process are considered to be critical for the final probiotic product’s efficacy and safety [32,33,34].

In conclusion, our study provides the first experimental evidence that Vivomixx^®^ may be a promising candidate against Crohn’s patients’ intestinal fibrosis. Of note, this property is not a common trait to the other probiotic VSL#3 formulation. Despite its unquestionable contribution to intestinal fibrosis, the TGF-β1/Smad signaling pathway inhibition could not be the only anti-fibrotic strategy that selectively blocks IBD-associated fibrosis. Therefore, it will be essential to analyze Vivomixx^®^’s ability to influence other potential molecular mechanisms that are implicated in the fibrotic cascade and immune-regulatory pathways crucial to intestinal mucosal homeostasis.

## Data Availability

All relevant data are within the manuscript.
